# Systemic Lupus Erythematosus

**DOI:** 10.1155/2012/815753

**Published:** 2012-12-05

**Authors:** Hiroshi Okamoto, Ricard Cervera, Tatiana S. Rodriguez-Reyna, Hiroyuki Nishimura, Taku Yoshio

**Affiliations:** ^1^Minami-Otsuka Clinic, Minami-Otsuka Institute of Technology, 2-41-9 Minami-Otsuka, Toshima-ku, Tokyo 170-0005, Japan; ^2^Department of Autoimmune Diseases, Hospital Clinic, Calle Villarroel, 170, Barcelona, Catalonia 08036, Spain; ^3^Department of Immunology and Rheumatology, National Institute of Medical Sciences and Nutrition Salvador Zubirán, Vasco de Quiroga 15, Colonia Sección XVI, 14000 Mexico City, DF, Mexico; ^4^Faculty of Biomedical Engineering, Toin University of Yokohama, 1614 Kurogane-machi, Aoba-ku, Yokohama 225-8802, Japan; ^5^Division of Rheumatology and Clinical Immunology, School of Medicine, Jichi Medical University, 3311 Yakushiji, Shimotsuke, Tochigi 329-0498, Japan

Systemic lupus erythematosus (SLE) is an autoimmune disease characterized by widespread immunologic abnormalities and multiorgan involvement including the skin, joints, and kidney as well as the peripheral and central nervous systems. The clinical course of SLE is usually dependent on the organ involved such as neuropsychiatric syndromes of systemic lupus erythematosus (NPSLE), lupus nephritis (LN), lupus pancreatitis (LP), and pulmonary hypertension (PH). Early diagnosis and epoch-making therapeutic strategies are prerequisites for improved prognosis of patients with SLE. To fulfill this medical need, a better understanding of the pathogenesis of SLE is required. Research over the course of several years has yielded a basic scientific understanding of SLE pathogenesis. This includes understanding the role of various cytokines and chemokines in disease progression, the chronic activation of plasmacytoid dendritic cells by circulating immune complexes, and the expression of neutrophil-specific genes.

The main focus of this special issue is on the diagnostic tools for organ involvement in SLE and in the development of new therapeutic strategies as well as the basic understanding of SLE pathogenesis. Thankfully, we have several research articles as well as outstanding review articles on this subject. We hope this special issue is an international forum for researchers and clinicians to summarize the most recent developments and ideas in the field.

To understand the pathogenesis of SLE, genetic background, several small molecules, and autoantigens/autoantibodies are extensively studied. In this special issue, we have several articles to introduce the recent topics in this field.

The extracellular matrix (ECM) is not only a structural support that maintains the architecture of tissues and organs, but also plays critical roles during inflammatory processes. Hyaluronan (HA) is a major component of the ECM that can directly regulate inflammatory processes through its interaction with its cell surface receptor CD44. In this special issue there is an outstanding review on the pathogenesis of SLE. S. Yung and T. M. Chan overviewed and discussed the putative mechanisms through which hyaluronan and its cell surface receptor, CD44, contribute to the pathogenesis of SLE, with particular emphasis on lupus nephritis.

Recently, the genetic background of SLE was revealed by a series of genome-wide association studies (GWAS) and several SLE susceptive genes including *MHC*, *BLK*, *ITGAM*, *STAT4*, *IRF5*, *BANK1*, and *ETS1* were identified. The polymorphisms on TNF*α*-induced protein 3 (TNFAIP3 or A20) interacting protein1 (TNIP1) have been reported to associate with the disease risk of several autoimmune diseases including SLE in Caucasians. J. Zhang et al. investigated the association of TNIP1 with SLE in the Chinese population using high-resolution melting (HRM) analysis. Although they found no association between TNIP1 SNPs with the disease risk of SLE in the Chinese population, they found a new SNP rs79937737 located on 5 bp upstream of rs7708392.

SLE is characterized by diverse clinical symptoms and autoantibody production against a variety of nuclear and cytoplasmic antigens. The occurrence and prevalence of these autoantibody specificities have been used to characterize the diverse clinical presentations of SLE. The standard screening assay for the detection of autoantibodies, immunodiffusion, and enzyme-linked immunosorbent assays (ELISAs) are often employed. R. Lu et al. introduced newer screening technologies, like the Luminex bead-based assay performed with the Bio-Rad BioPlex 2200, which focus on performing a sensitive multiplex analysis of selecting autoantibody specificities. They studied the association between autoantibody specificities and SLE ACR criteria and subcriteria using conditional logistic regression analysis with sera from European American SLE patients.

I. Aganovic-Musinovic et al. evaluated the concentration values of each antigen of extractable nuclear antigens (ENA)-6 profiles to investigate possible correlation between the concentration of Sm antibodies and circulating immune complexes. Based on calculations from ROC curves, they found that Sm/RNP was clearly a very important marker for diagnosis of SLE.

In lupus nephritis (LN), autoantibodies have been shown to be critical in the initiation and progression of renal injury, via interactions with both Fc-receptors and complement. One approach in the management of patients with LN is the use of intravenous immunoglobulin. This therapy has shown benefit in the setting of many forms of autoantibody-mediated injury; however, the mechanisms of efficacy are not fully understood. S. E. Wenderfer and T. Thacker overview the data supporting the use of intravenous immunoglobulin therapy in LN as well as discuss the potential mechanisms of action.

In this special issue, we have excellent reviews on various manifestations of SLE, such as ocular manifestations, cutaneous manifestations, osteonecrosis as well as vascular diseases, a complication of SLE, and neonatal lupus, a specific form of lupus.

About one-third of patients with SLE have ocular manifestations, such as keratoconjunctivitis sicca, retinal vasculitis, and optic neuritis/neuropathy. Prompt diagnosis and treatment of eye disease are paramount as they are often associated with high levels of systemic inflammation and end-organ damage. N. V. Palejwala et al. clearly reviewed the mechanism, diagnosis, and treatment of ocular manifestations in SLE with a large collection of clinical pictures. They also discuss the use of immunosuppressive agents as well as biologic agents. This review provides a great lesson for all the physicians who treat SLE patients, especially rheumatologists.

The skin is one of the target organs most variably affected and is involved in up to 85% of SLE cases. Cutaneous lesions account for four of these 11 criteria of SLE established by the American College of Rheumatology (ACR). Skin lesions in patients with lupus may be specific or nonspecific. L. Uva et al. clearly reviewed the SLE-specific cutaneous changes: malar rash, discoid rash, photosensitivity, and oral mucosal lesions as well as SLE nonspecific skin manifestations, their pathophysiology, and management. This review includes typical pictures of various types of cutaneous lesions and also provides us a great lesson to learn.

Vascular disease is frequent in patients with SLE and represents the most frequent cause of death in established disease. A. Pyrpasopoulou et al. overviewed the prevalence of the different forms of vasculopathy that can be encountered in a lupus patient, described their pathogenesis, and addressed their impact on disease severity and outcome.

Osteonecrosis may complicate the course of SLE and may contemporaneously affect multiple joints. The major risk factor associated with the development of osteonecrosis is the use of glucocorticoid at high doses. P. Caramaschi et al. discuss the pathogenesis of osteonecrosis in SLE patients, factors associated with the development of osteonecrosis, relationship between osteonecrosis development, and type of corticosteroid use as well as the treatment of osteonecrosis in SLE.

Neonatal lupus erythematosus (NLE) refers to a clinical spectrum of cutaneous, cardiac, and systemic abnormalities observed in newborn infants whose mothers have autoantibodies against Ro/SSA and La/SSB. The condition is rare and usually benign and self-limited but sometimes may be associated with serious sequelae. K. L. Hon and A. K. C. Leung review the pathophysiology, clinical features, and management of infants with this condition. They also provide us the evidence of the prognosis of NLE and also propose a question about the management of future pregnancies of SLE women.

We hope that readers of Autoimmune Diseases will find in this special issue not only attractive data, but also useful reviews on crucial aspects of SLE.

*Hiroshi Okamoto* *Ricard Cervera* *Tatiana S. Rodriguez-Reyna* *Hiroyuki Nishimura* *Taku Yoshio*

